# Factors associated with persistent blood stream infection in the Neonatal Intensive Care Unit

**DOI:** 10.21203/rs.3.rs-6946297/v1

**Published:** 2025-07-01

**Authors:** Hanna Lee, Noa Fleiss, Matthew Bizzarro, Richard Feinn, Michelle Rychalsky, Christine Puthawala, David R. Peaper, Thomas S. Murray

**Affiliations:** a Department of Pediatrics, Yale School of Medicine, New Haven, CT.; b Department of Biomedical Sciences, Frank H Netter MD School of Medicine, Quinnipiac University; c Department of Pharmacy, Yale New Haven Children’s Hospital, New Haven CT; d Department of Laboratory Medicine, Yale School of Medicine, New Haven, CT.

## Abstract

**Objective:**

This study determined factors associated with persistent blood stream infections (BSIs) for infants in the NICU to identify when follow up blood cultures (FUBCs) have increased utility.

**Study design:**

Single center study of all infants in a level IV NICU (n=121) with a positive blood culture over a five-year period. Clinical and microbiological variables were examined with bivariate and multi-regression analyses to identify factors associated with persistent BSI, defined as growth of the same organism >48 hours after the index culture.

**Results:**

The recovery of *Staphylococcus aureus* (OR=6.10, p<.001), male sex (OR=3.31, p=.020), the presence of a central venous catheter (OR=3.73, p=.020), and BSI in the setting of late-onset sepsis (p<.001) were associated with persistent BSI. No infants with either early-onset sepsis or growth of Streptococcal sp. had a persistent BSI.

**Conclusion:**

In the NICU, both patient and microbial characteristics can inform diagnostic stewardship regarding the need for FUBCs.

## Introduction

Bloodstream infections (BSIs) in premature infants are a significant cause of morbidity and mortality in this high-risk population (1, 2). In premature infants with clinical signs of infection, the evaluation often entails obtaining two separate site blood cultures, with a minimum of 1.0 ml of blood per bottle, although guidance can vary among different references (3–6). Drawing blood from premature infants is challenging given the size and caliber of their vessels (7). Multiple attempts at collecting blood for culture can lead to contamination and increased antibiotic use (8). Unnecessary blood collection should also be avoided due to infants’ lower circulating blood volume compared with older children and adults (9). One aspect of diagnostic stewardship is the practice of avoiding tests when the pre-test probability of a clinically actionable result is low. Although the approach can vary, criteria exist for when to draw blood cultures for infants in the neonatal intensive care unit (NICU) as part of a sepsis evaluation, (3, 10). After an initial negative blood culture, the clinical utility of frequent follow-up blood cultures (FUBCs) is low (11). However, there is less information for infants in the NICU about when to draw FUBCs after an index positive result.

In the adult population, indications for FUBCs after a positive blood culture include an endovascular focus, the presence of a central venous catheter (CVC) or the recovery of *Staphylococcus aureus* or yeast (12–14). FUBCs are generally not recommended for BSI attributed to Gram negative rods (GNR) or Streptococcal species (12, 15, 16). In hospitalized children outside of the NICU, studies vary regarding the need for FUBCs after GNR bacteremia (17–19). Similar to adults, we recently showed no positive FUBCs from children that initially grew any Streptococcal species including *S. agalactiae* (Group B streptococcus, GBS) as well as a higher likelihood of persistent bacteremia in the setting of *S. aureus*, yeast, the presence of a CVC, and a history of a previous BSI (18). At our institution, repeat blood cultures are common for any child after a positive blood culture result regardless of the organism or clinical suspicion of BSI. Given the lack of data on FUBCs after an index positive for infants in the NICU, we examined the risk factors for persistent bacteremia to inform clinical decisions regarding the need for FUBC.

## Methods

### Setting and Patient population:

We conducted a single center cohort study from August 1, 2016-December 31, 2021, at the Yale New Haven Children’s Hospital (YNHCH) Level IV NICU, a tertiary referral center supporting a high-risk delivery network. From 1992–2018, the 54-bed YNHCH NICU had an open bay design with multiple infants sharing a large space. In January 2018, the NICU moved to a new location incorporating 68-beds comprised mostly of single rooms. Most NICU admissions are inborn with approximately 15% transferred in from regional community hospitals after delivery. Once infants are discharged from the NICU, subsequent readmissions are to a pediatric unit.

Initial inclusion criteria for this investigation included any infant admitted to the YNHCH NICU from August 1, 2016- December 31, 2021, with a positive blood culture. Persistent BSI was defined as a FUBC that was positive for the same organism >48 hours after the time of the initial blood culture collection. Infants were excluded if they died prior to 48 hours after the initial positive culture and a FUBC could not be obtained. A new positive blood culture obtained greater than seven days after the initial positive or recovery of a different organism from the FUBC was considered a new BSI. This protocol was approved by the Yale Investigations Review Board with a waiver of consent (#2000032485).

### Blood culture collection and clinical microbiology:

Blood cultures were collected via sterile technique, using povidone iodine as an antiseptic for skin disinfection prior to arterial stick or venipuncture. For blood cultures from CVCs, the hub was wiped with either 70% isopropyl alcohol or a 3.15% chlorhexidine gluconate/70% isopropyl alcohol product per the manufacturer’s instruction for use. Blood culture guidelines at our institution state that unless anaerobic infection is strongly suspected, blood should be drawn from two separate sites and inoculated into two aerobic adult blood culture bottles. Our laboratory does not use pediatric bottles. Our institution follows the recommendations from the 2018 American Academy of Pediatrics (AAP) guidelines, recommending that at least 1 ml of blood be obtained per blood culture bottle to reliably detect bacteremia (3). Blood culture volumes were not routinely monitored for this unit during the study period.

Details on the work up of positive blood cultures by the clinical microbiology laboratory during this period have been previously published (18). Briefly, the on-site clinical microbiology laboratory uses the BD BacTec blood culture system (Becton, Dickinson Life Sciences) with positive results subject to Gram stain followed by the inoculation of appropriate agar plates. In 2017 the MRSA/SA Blood Culture Assay (Cepheid, USA) was introduced for positive blood cultures demonstrating Gram positive cocci in clusters. Identification of organisms after bacterial growth was primarily performed by matrix assisted laser-time of flight mass spectrometry (MALDI-TOF MS; Vitek MS, Biomerieux) supplemented with antigen or biochemical methods for Staphylococcal species or those failing MALDI-TOF MS identification. Antibiotic susceptibility testing was performed per the Clinical Laboratory Standards Institute guidelines at the time of the culture and included microbroth dilution (Vitek 2, Biomerieux), disk diffusion, or E-test depending on the organism and antibiotic combination. *Data collection*: Yale New Haven Hospital has tracked all positive blood cultures in the newborn population since 1928 (20). BSI were categorized by timing as early-onset sepsis (EOS), defined as a BSI detected at ≤3 days of life, and late-onset sepsis (LOS) as one detected at >3 days. Evaluation and treatment for suspected EOS was based largely on the presence of maternal risk factors (e.g., chorioamnionitis, prolonged rupture of amniotic membranes) and, for LOS, on clinical signs of infection including apnea, bradycardia, temperature instability, glucose instability, lethargy, and hypotension requiring intervention. Data from August 1, 2016-December 31, 2021, was cross checked with information extracted from the electronic medical record by the Yale Data Analytics Team using the inclusion criteria above. Manual chart review confirmed demographic and clinical variables, determined appropriate empiric antibiotic use and whether the recovered organism was a likely pathogen or contaminant. Empiric antibiotics at the time of blood culture draw were considered appropriate if either the organism is universally considered susceptible to an antibiotic (e.g., vancomycin for *S. aureus*, ampicillin for GBS) or chart review confirmed the organism was susceptible to any empiric antibiotic started at the time of index culture. Criteria for a likely contaminant were defined as a single positive blood culture not treated for >5 days that yielded either an organism not commonly associated with neonatal sepsis, or the culture was polymicrobial. Potential contaminants were included to assess the results of FUBCs for these isolates. Aggregate, deidentified data are available by contacting the corresponding author.

### Statistics:

Descriptive statistics are presented at the patient and specimen level and included frequencies with percentages for categorical variables and medians with interquartile range for quantitative variables. To test for demographic and clinical factors associated with persistent BSI generalized estimating equations with a binomial distribution and logit link was run separately for each factor. The model included unstructured covariance with robust standard errors. This was followed with a backward stepwise multivariate model to determine the combination of factors that predicted persistent BSI. Sensitivity analysis was performed using the multivariate model removing potential contaminants. Factors with p-values <0.05, as determined by bootstrap sampling, were retained in the final multivariate model.

## Results

### Patient demographics.

**Figure 1.**
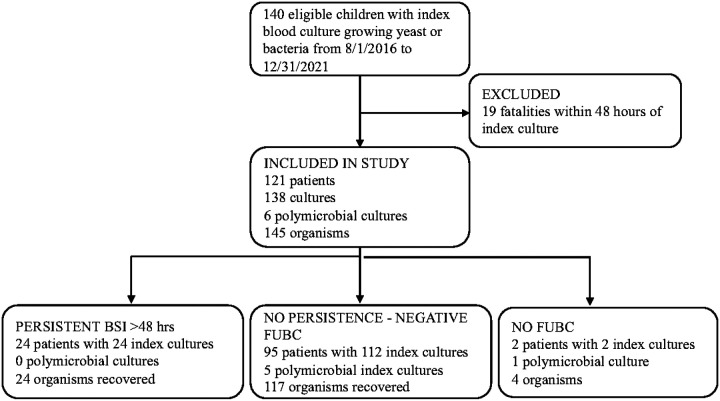
Flow diagram of positive blood cultures in the Neonatal Intensive Care Unit during the study period BSI= blood stream infection, FUBC= follow up blood culture.

One hundred and forty infants had a positive blood culture during the study period with 19 meeting exclusion criteria (Figure 1). The 121 remaining infants had 138 positive index blood cultures (Figure 1). In our study cohort, 99% (136/138) of specimens were followed with a second blood culture, including 95% (19/20) of blood cultures where the original organism(s) was classified as a likely contaminant, none of which grew an organism on FUBC. The two infants without FUBC were not considered persistent BSI. 17% (24/138) of positive index cultures were persistently positive on FUBC (Figure 1). Index cultures that grew likely contaminants represented 15% (20/138) of the specimens. Six FUBCs grew a new index organism, all of which were coagulase negative Staphylococci that subsequently were not persistent after further FUBCs.

The demographics of the patient population are shown in Table 1. The median gestational age was 29 weeks [IQR 25–37]; 26 weeks for infants with persistent BSI compared with 30 weeks for those without. The median white blood cell count for the entire cohort was 12,900/ml [IQR 7,800/ml −21,300/ml]; 14,300/ml in infants with persistent BSI compared with 12,100/ml in infants without persistent BSI. The median platelet count for the entire study group was 219,000/ml [IQR 106,000/ml −300,000/ml]; 131,000/ml for infants with persistent BSI and 230,000/ml for those without. All positive FUBCs occurred in infants being evaluated and treated for LOS.

### Microbiology.

For 19 infants, persistent BSI could not be determined because of death within 48 hours of the index culture; these infants were excluded from further analysis. These BSIs were caused by a variety of Gram negative and Gram positive organisms (Supplementary Table 1). The recovered microbes from initial cultures from eligible patients are shown in Table 2. Consistent with literature from adults and older children, the most common organism associated with persistent BSI was *S. aureus*, which accounted for 63% (15/24) of all persistent BSI. In fact, 41% (15/37) of *S. aureus* BSIs remained positive at 48 hours, despite appropriate empiric antibiotics for 93% (14/15) of these infants (Table 2, Supplementary Table 2). There were four infants with a MRSA BSI, all treated with appropriate empiric antibiotics. Fifty percent (2/4) of MRSA were persistent. BSI caused by Enteric Gram negative rods were recovered in 12% (5/46) of FUBC (Table 2). *Klebsiella* sp. caused persistent BSI in 20% (2/10) of infants while *Escherichia coli* was recovered in 8% (2/26) of FUBCs (Table 2, Supplementary Table 2). No Streptococcal species (n=18) were recovered in any FUBC (Table 2). Of the organisms without available antibiotic susceptibility data or treated empirically with inappropriate antibiotics, of which 14 were considered likely contaminants, the only persistent organism (1/27, 4%) was a single *S. aureus*. 23 of the 24 persistent organisms (96%) were empirically started on an antibiotic that the organism was susceptible to, demonstrating persistence regardless of appropriate therapy (Supplementary Table 2).

### Host and microbial factors associated with persistent BSI.

Bivariate analyses revealed that the following factors were significantly associated with persistence: male sex, LOS (>3 days after birth; as compared with EOS), presence of CVC, any type of respiratory support, post-natal steroid exposure within 48 hours of the index culture, and recovery of *S. aureus* from the index culture (Table 2 and Supplementary Table 3).

Since there are several potential confounding variables related to prematurity, a multivariate model was constructed. In this model, factors that remained significantly associated with persistent BSI included LOS, recovery of *S. aureus*, presence of a CVC, and male sex (Table 3). We included blood cultures with contaminants because we were interested in how many received FUBC and whether new organisms were isolated. However, the inclusion of probable contaminants, with an increased proportion of coagulase negative Staphylococci, could impact the analysis of factors associated with persistent BSI. Therefore, we performed sensitivity analysis removing the likely contaminants and there were no changes to the results of the multivariable model (data not shown). Given the higher rates of persistence in smaller babies, we also re-analyzed the data comparing all infants above and below 1500 grams birth weight and this also did not change the results of the model.

## Discussion

Collecting blood from premature infants is challenging. Informed diagnostic stewardship that safely reduces the number of blood draws in the NICU population is possible and can reduce patient harm (20). While there are clear indications in the adult population regarding FUBCs after an index positive culture, data to help guide this decision for infants in the NICU are lacking. In this cohort, 99% of initial positive blood cultures were followed with a blood culture >48 hours later, including 95% of likely contaminants. This suggests an opportunity to safely reduce the number of FUBCs and associated needle sticks. Like literature for adults and older children, the recovery of *S. aureus* and the presence of a CVC were both associated with a higher rate of persistence (12, 18). Infants with either of these risk factors require a FUBC to document clearance and help determine length of antibiotic therapy. Given the prevalence of *S. aureus* invasive infections in NICUs, there is a need for continued efforts to understand the epidemiology and reduce invasive disease for both MSSA and MRSA in the NICU setting (21–23). Consistent with previous studies of adults and children outside of the NICU, no Streptococci of any species were recovered from FUBCs (15, 18). If confirmed with larger data sets, this may offer an opportunity for diagnostic stewardship by reducing FUBCs in infants with Streptococcal bacteremia.

LOS was determined to be a significant predictor of persistent bacteremia as compared with EOS and, surprisingly, no infant with EOS (n=39 specimens) had a persistent BSI. In the NICU population, the decision to evaluate and initiate empiric treatment for EOS is largely based on risk factor assessment, including gestational age and the presence of maternal risk factors for neonatal infection (3, 24). In neonates <35 weeks’ gestation, given their increased risk of infection and associated morbidity and mortality, assessment and intervention occurs quickly after birth and often in the absence of clear clinical signs of infection. Alternatively, the decision to evaluate and treat for LOS is based almost exclusively on a change in baseline condition and the presence of clinical and/or laboratory signs of infection. A prior study on the use of polymerase chain reaction as part of the evaluation for suspected neonatal infection determined a positive correlation between higher bacterial loads in the blood and the presence of a higher number of clinical signs of infection (25). It has also been speculated that intrapartum antibiotic exposure may lead to lower bacterial levels in neonates with EOS (26). In our study population with confirmed EOS, only 17% had clinical signs of infection at the time of the evaluation (as compared with 100% of those with LOS), and 71% of infants evaluated for EOS were delivered in the setting of intrapartum antibiotics administration (data not shown). It is therefore possible that higher bacterial loads in infants with LOS as compared with infants with EOS contributed to the significantly higher observed odds of persistent BSI.

The pathophysiology of LOS is distinct from EOS and may also contribute to persistent BSI. Unlike EOS, which originates from ascension of maternal genitourinary tract microbes during labor or rupture of membranes, LOS is usually a hospital-acquired infection from exposure to invasive procedures or other environmental sources (27–29). Pathogen exposure in LOS can occur due to contamination of indwelling medical devices, such as a CVC or an endotracheal tube. This contrasts with EOS, where pathogen exposure originates from the mother and exposure is removed once the infant is delivered (27).

A near equal percentage of male and female infants in our study population had an initial positive blood culture, yet male sex was associated with significantly higher odds of a positive FUBC. This finding aligns with observed gender disparities in the preterm population, where several studies have demonstrated higher rates of morbidity and mortality among male preterm infants compared with their female counterparts (25, 30, 31). Of the 19 infants that died within 48 hours of a positive index culture, 13 (68%) were male (Supplementary Table 1). A 2022 study investigated the association between sex and long-term pediatric infectious disease morbidity in a cohort of 2111 dichorionic twin pairs followed up to 18 years of age. A higher risk of ear, nose and throat, central nervous system, and respiratory infections were observed in male twins as well as a higher cumulative hazard of infectious disease-related morbidity and hospitalization as compared with females (32). Although the precise mechanism of these differences remains unknown, gender-specific immunologic, genetic, and hormonal differences have been hypothesized to contribute (31). To our knowledge, an association between male sex and persistent BSI has not been previously observed.

There are limitations that require additional study. As a single center study, the number of persistent BSIs were low and there were few Candida sp. BSIs, an organism that requires FUBC to determine treatment length (13). We also did not routinely measure blood volume received for culture during the study period and our laboratory only stocks adult blood culture bottles. Thus, it is possible there were missed episodes of persistent BSI if the FUBC did not have sufficient volume to detect growth.

## Conclusions

In this single center study, *S. aureus* bacteremia, the presence of a CVC, male sex and late-onset sepsis were associated with persistent BSI in infants in the NICU, suggesting clinical utility of documenting clearance with FUBCs. For early-onset sepsis and Streptococcal bacteremia, no persistent BSIs were identified and, if validated in larger studies, offer an opportunity for diagnostic stewardship.

## Supplementary Material

Supplementary Files

This is a list of supplementary files associated with this preprint. Click to download.

- SupplementaryTable1final.pdf
- SupplementaryTable2final.pdf
- SupplementaryTable3final.pdf

## Figures and Tables

**Table 1.** Characteristics of infants with positive blood cultures in the NICU

	Overall (%)	Persistent BSI n (%)	No Persistent BSI n (%)
Patient Level	121 (100)		
Sex			
Female	61 (50)	8 (13)	53 (87)
Male	60 (50)	16 (27)	44 (73)
Birthweight			
<1000g	49 (41)	14 (29)	35 (71)
1000–1499g	15 (12)	3 (20)	12 (80)
1500–2499g	16 (13)	2 (13)	14 (87)
≥2500g	41 (34)	5 (12)	36 (88)
Structural congenital cardiac disease	21 (17)	4 (19)	17 (81)

Specimen Level	138 (100)	24 (17)	114 (83)
Single organism on Index Culture	126 (92)		
New organism on FUBC	6 (4)	24 (19)	102 (81)
Polymicrobial Index culture	6 (4)	0 (0)	6 (100)
Onset of Sepsis			
Early	39 (28)	0 (0)	39 (100)
Late	99 (72)	24 (24)	75 (76)
Appropriate antibiotics			
No	16 (12)	1 (6)	15 (94)
Yes	111 (80)	23 (21)	88 (79)
Missing^a^	11 (8)	0 (0)	11 (100)
Central venous catheter			
No	71 (51)	6 (8)	65 (92)
Yes	67 (49)	18 (27)	49 (73)
Respiratory support^b^			
No	36 (26)	2 (6)	34 (94)
Yes	102 (74)	22 (22)	80 (78)
Organism recovered from another site^c^			
No	90 (65)	12 (13)	78 (87)
Yes	48 (35)	12 (25)	36 (75)
Steroids within 48 hours of index blood culture			
No	126 (91)	19 (15)	107 (85)
Yes	12 (9)	5 (42)	7 (58)

FUBC= follow up blood culture

a Ten Coagulase negative staphylococcus and one Bacillus sp.

b Mechanical ventilation/Continuous positive airway pressure/Noninvasive positive pressure ventilation/Humidified high flow nasal cannula

c Some infants had positive cultures/evidence of infection from multiple sites [gastrointestinal (20), skin/soft tissue (12), pulmonary (11), urine (5), central nervous system (3), intravascular (2)

**Table 2.** Microbiology of infants with positive blood cultures in the NICU

	No. (%)			
Characteristic	Overall	Not persistent BSI	Persistent BSI	P value
All organisms	145	121 (83.4)	24 (16.6)	<0.001
Gram positive organisms				
*Staphylococcus aureus*^a^	37 (25.5)	22 (59.5)	15 (40.5)	
*Coagulase negative staphylococci*	29 (20.0)	27 (93.1)	2 (6.9)	
Gram positive rods^b^	4 (2.8)	4 (100)	0	
*Enterococcus* species	7 (4.8)	6 (85.7)	1 (14.3)	
*Streptococcus agalactiae*	10 (6.8)	10 (100)	0	
*Viridans group Streptococcus*^c^	8 (5.5)	8 (100)	0	
*Micrococcus* species	1 (0.7)	1 (100)	0	
Gram negative organisms				
Enteric^d^	42 (29)	37 (88)	5 (12)	
Non-glucose fermenting^e^	3 (2.1)	3 (100)	0	
Diplococci^f^	2 (1.4)	2 (100)	0	
*Candida parapsilosis*	2 (1.4)	1 (50)	1 (50)	

a Includes methicillin-sensitive (4) and methicillin-resistant *S. aureus* (33)

b *Bacillus* species, *Corynebacterium* species, *Listeria monocytogenes*, gram positive rods not specified

c Includes *S. anginosus*, *S. gallolyticus*, *S. mitis*, *S. sanguinis*

d Citrobacter species (1), Pluralibacter (Enterobacter) species (1), *Escherichia coli* (26), Klebsiella species (10)*, Serratia marcescans*(2), *Pantoea agglomerans* (1), *Proteus mirabilis* (1)

e Acinetobacter species (1), *Pseudomonas aeruginosa* (2).

f Neisseria mucosa, Moraxella osloensis

**Table 3:** Variables associated with persistent blood stream infection

Predictor	Coefficient	Standard Error	Odds Ratio	95% Confidence interval	P-Value
Late-onset sepsis	19.31	0.44	>100	-	<.001
*S. aureus*	1.72	0.63	5.59	1.93 – 16.19	.003
Central venous catheter	1.34	1.06	3.81	1.25 – 11.56	.018
Male	1.12	0.55	3.05	1.04 – 8.93	.042

These results are from a Multivariate Model with Bootstrap Confidence Intervals
